# Network Pharmacology-Based Study on the Mechanism of *Scutellariae Radix* for Hepatocellular Carcinoma Treatment

**DOI:** 10.1155/2020/8897918

**Published:** 2020-10-26

**Authors:** Qian Wang, Yan Liang, Can Peng, Peng Jiang

**Affiliations:** ^1^Department of Pharmacy, Anhui University of Chinese Medicine, Hefei 230012, China; ^2^Institute of Pharmaceutics, Anhui Academy of Chinese Medicine, Hefei 230012, China; ^3^Anhui Province Key Laboratory of Pharmaceutical Formulation Technology and Application, Anhui University of Chinese Medicine, Hefei 230012, China; ^4^Department of Pharmacy, Division of Life Sciences and Medicine, The First Affiliated Hospital of USTC, University of Science and Technology of China, Hefei 230031, China

## Abstract

Hepatocellular carcinoma (HCC) is a malignant tumor without effective therapeutic drugs for most patients in advanced stages. *Scutellariae Radix* (SR) is a well-known anti-inflammatory and anticarcinogenic herbal medicine. However, the mechanism of SR against HCC remains to be clarified. In the present study, network pharmacology was utilized to characterize the mechanism of SR on HCC. The active components of SR and their targets were collected from the traditional Chinese medicine systems pharmacology database and the traditional Chinese medicine integrated database. HCC-related targets were acquired from the liver cancer databases OncoDB.HCC and Liverome. The gene ontology and the Kyoto Encyclopedia of Genes and Genomes pathway were analyzed using the Database for Annotation, Visualization, and Integrated Discovery. Component-component target and protein-protein interaction networks were set up. A total of 143 components of SR were identified, and 37 of them were considered as candidate active components. Fifty targets corresponding to 29 components of SR were mapped with targets of HCC. Functional enrichment analysis indicated that SR exerted an antihepatocarcinoma effect by regulating pathways in cancer, hepatitis B, viral carcinogenesis, and PI3K-Akt signaling. The holistic approach of network pharmacology can provide novel insights into the mechanistic study and therapeutic drug development of SR for HCC treatment.

## 1. Introduction

Hepatocellular carcinoma (HCC) is the fifth most common and lethal malignancy in the world [1]. It is a complex disease closely linked to chronic viral infection, carcinogenesis of toxins, and cirrhosis induced by fatty liver disease or alcohol abuse and genetic factors [2]. Several common therapeutic options for HCC are currently considered, including surgical resection, local ablation, transarterial chemoembolization, liver transplantation, and systemic treatment with sorafenib [3]. Sorafenib is a kinase inhibitor drug approved for the treatment of primary kidney cancer, advanced primary liver cancer, FLT3 internal tandem duplication (FLT3-ITD) positive acute myeloid leukemia (AML), and radioactive iodine resistant advanced thyroid carcinoma. However, a majority of patients with HCC are diagnosed at advanced stages, and the only feasible treatment for these patients is sorafenib [4]. Furthermore, less than 20% of patients respond well to sorafenib that often causes serious adverse reactions [5]. Therefore, more effective alternative therapies with low toxicity should be developed to improve the overall survival of patients with HCC.

Chinese herbal medicine (CHM) has been used clinically for thousands of years. With advanced scientific evaluation in basic research and clinical trials [6], CHM is recognized as a treasure house for alternative anticancer drug development [7]. Extensive research has shown that some specific herbs and natural compounds can effectively inhibit cell proliferation, interfere with tumor progression, and block tumor metastasis [8, 9]. *Scutellariae Radix* (SR) is the root of plant Scutellaria baicalensis Georgi (Lamiaceae). SR is a well-known anti-inflammatory and anticarcinogenic herbal medicine [10, 11], which can prevent and treat liver injuries caused by hepatitis and liver cancer. SR has certain components, such as baicalin and baicalein, which show an evident antitumor effect on hepatocarcinoma [12–14]. However, the mechanism of antitumor action of SR against HCC is still unclear.

As an emerging discipline in modern CHM pharmacological research, network pharmacology has been successfully undertaken to screen active components and reveal the pharmacodynamic mechanisms of CHM [15]. In contrast to Western medicine based on one drug and one target, CHM exerts its pharmacological action as a whole through multicomponents and multitargets. With the holistic perspective of CHM, network pharmacology intends to investigate the effects of drugs on disease at a holistic level [16]. It changes the research strategy from the existing “one drug, one target” mode to the emerging “one drug, network targets” mode [17] or “multicomponents, network targets” mode [18]. This approach is feasible for investigating the mechanisms of the effect of CHMs and their synergistic actions in cancer treatment [19]. To the best of our knowledge, only two network pharmacology reports are associated with SR, i.e., SR against diabetes mellitus [20] and baicalein on HCC [17]. However, network pharmacology evaluation has yet to be performed to determine the molecular mechanism of SR for HCC treatment.

**Figure 1:**
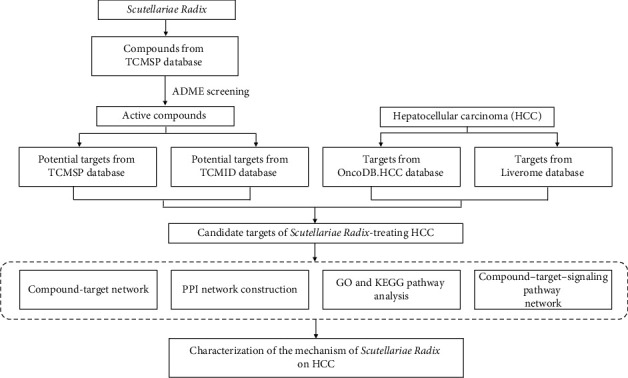
Flowchart of designed analysis in *Scutellariae Radix* against hepatocellular carcinoma.

The present study applies computer simulation and bioinformatic data mining to build a pharmacological network of SR for HCC treatment and search for the candidate bioactive components, protein targets, and pathways. Network pharmacology provides a potent and promising approach for the application and development of CHM to HCC therapy (Figure 1).

## 2. Methods

### 2.1. Active Components in SR

All herbal medicinal ingredients of SR were obtained from the traditional Chinese medicine systems pharmacology database (TCMSP; http://tcmspw.com/tcmsp.php) [21], which is a unique analysis platform of systems pharmacology contributing to drug discovery from herbal medicines. The candidate active components of SR were obtained on the basis of the criteria of drug likeness (DL) of ≥0.18 and oral bioavailability (OB) of ≥30%, which are the principal properties to determine the drug ability of compounds.

### 2.2. Prediction of Drug Targets for SR

The human protein targets of the active components of SR were obtained from the traditional Chinese medicine integrated database (TCMID), http://www.megabionet.org/tcmid/) and the TCMSP database [21]. The TCMID database is a comprehensive database to offer a reference and build bridges between traditional Chinese medicine and current medicine. The gene symbol names were further clarified with their UniProt ID from the UniProtKB database (http://www.uniprot.org), which is a unique protein database partially revised by experts.

### 2.3. Candidate Targets of SR for HCC Treatment

HCC-related genes were retrieved from Liverome (http://liverome.kobic.re.kr/index.php) [22] and OncoDB.HCC (http://oncodb.hcc.ibms.sinica.edu.tw) [23], which are two liver cancer-related databases. The drug targets of the candidate active components of SR were mapped to HCC-related targets to obtain the candidate targets of SR for HCC treatment. The network between the components and corresponding targets was constructed and visualized using the Cytoscape software 3.7.2 (http://www.cytoscape.org/).

### 2.4. Protein-Protein Interaction Network Construction and Analysis

The candidate targets of SR for HCC treatment were imported into the STRING database (Version 11.0) [24] (https://string-db.org/) to construct a protein-protein interaction (PPI) network. The criteria settings were set as follows: high confidence score >0.7 and max number of interactors ≤60. Then, the results were imported into the Cytoscape software for visualization and further analysis. Network Analyzer [25], a Cytoscape plugin, was used to analyze the important network parameters of nodes and edges for constructing the PPI network of core targets.

### 2.5. Gene Ontology and Pathway Enrichment Analysis

The gene ontology (GO) and the Kyoto Encyclopedia of Genes and Genomes (KEGG) pathway enrichment were analyzed using DAVID [26] 6.8 (http://david.abcc.ncifcrf.gov). DAVID is an online functional annotation database to understand the biological meaning of a large list of genes. The results of GO and KEGG pathway analyses were visualized with GraphPad Prism 7.0 and OmicShare platform (https://www.omicshare.com/), respectively. The compound-target-signaling pathway network was comprehensively constructed using Cytoscape 3.7.2.

## 3. Results

### 3.1. Active Components of SR

A total of 143 components of SR were retrieved from the TCMSP database; among them, 37 components conformed to the screening standards of the DL index ≥ 0.18 and OB ≥ 30%. These 37 components were chosen as candidate active components, and the properties of the active components are shown in Table 1.

### 3.2. Prediction of the Drug Targets of SR

A total of 797 protein targets corresponding to the 37 candidate active components were obtained from the TCMID and TCMSP databases. A total of 291 targets and 506 targets were derived from the TCMSP and TCMID databases, respectively. The detailed information is shown in Supplementary Table S1. After the overlaps were removed, 368 protein targets were acquired for further analysis.

### 3.3. Candidate Targets of SR against HCC

**Figure 2:**
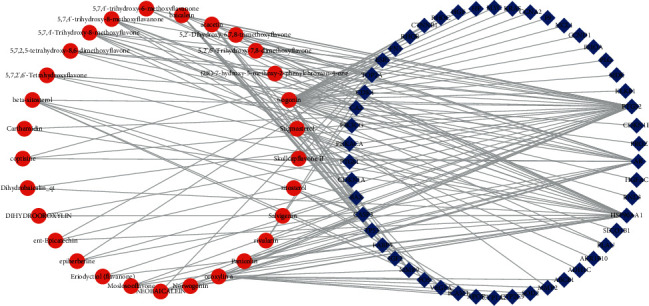
Compound-compound target network of *Scutellariae Radix* in the treatment of hepatocellular carcinoma. Red ellipse represents candidate active compounds and blue diamond represents potential protein targets.

A total of 566 HCC-related targets were obtained from OncoDB.HCC and Liverome databases. The detailed information of HCC-related targets is displayed in Supplementary Table S2. The targets of each candidate active component were mapped to HCC-related targets. After aggregation, 50 targets of 29 active components were acquired as candidate targets of SR for HCC treatment. The information of these 50 candidate targets is presented in Supplementary Table S3. The component-component target network of SR for HCC treatment was established (Figure 2), and it comprised 79 nodes (50 for candidate protein targets and 29 for potential active components). Of the 29 active components, 3 components, namely, wogonin (degree = 32), oroxylin A (degree = 12), and baicalein (degree = 11), and multiple HCC targets exerted high degree levels. Of the 50 protein targets, 3 that were linked to more components showed high degree levels, such as prostaglandin endoperoxide synthase 2 (PTGS2; degree = 28), heat shock protein HSP 90-alpha (HSP90AA1; degree = 25), and androgen receptor (AR; degree = 17). These candidate targets with a high degree might play an essential role in the treatment network of SR for HCC treatment.

### 3.4. PPI Network Construction and Analysis

**Figure 3:**
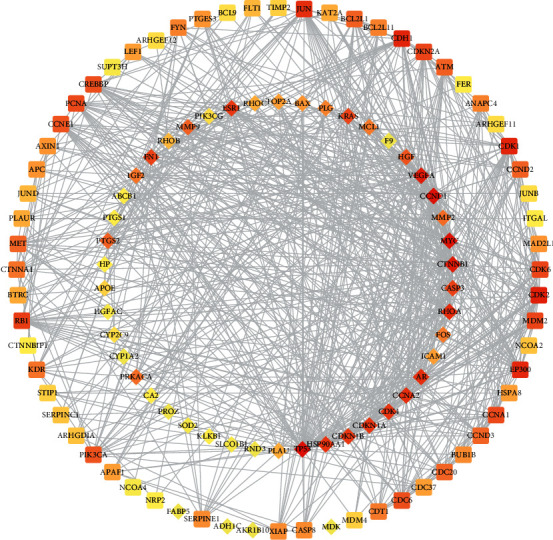
Protein-protein interaction network of the compound targets of *Scutellariae Radix* in the treatment of hepatocellular carcinoma. Diamond represents compound target and round rectangle represents human other protein.

**Figure 4:**
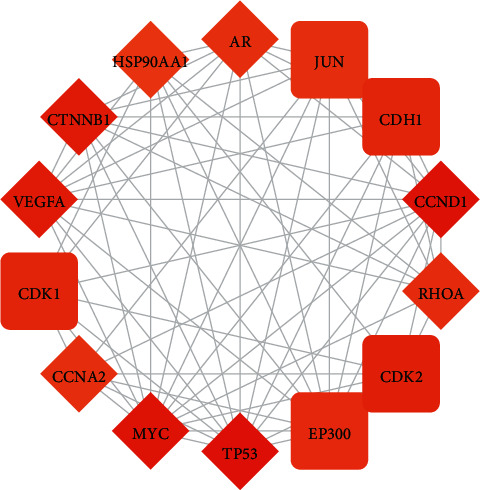
Hub targets of *Scutellariae Radix* in the treatment of hepatocellular carcinoma. Diamond represents compound target and round rectangle represents human other protein.

A PPI network was built to predict and illuminate the relationship between the candidate protein targets and other human proteins, with 110 nodes (50 candidate protein targets and 60 other human proteins that interacted closely with candidate protein targets) and 738 edges (Figure 3). The degree of nodes ranged from 1 to 53, and the mean degree was 13.906. The core targets (hub genes) were nodes whose degree was ≥27.812. The PPI network of the core targets was constructed (Figure 4). These targets included candidate protein targets such as cellular tumor antigen p53 (TP53), cyclin D1 (CCND1), myc protooncogene protein (MYC), vascular endothelial growth factor A (VEGFA), catenin beta-1 (CTNNB1), transforming protein RhoA (RHOA), cyclin-A2 (CCNA2), AR, and HSP90AA1, and human other targets including cyclin-dependent kinase 2 (CDK2), cadherin-1 (CDH1), cyclin-dependent kinase 1 (CDK1), E1A binding protein P300 (EP300), and transcription factor AP-1 (JUN).

### 3.5. GO and KEGG Enrichment Analysis of Candidate Targets

**Figure 5:**
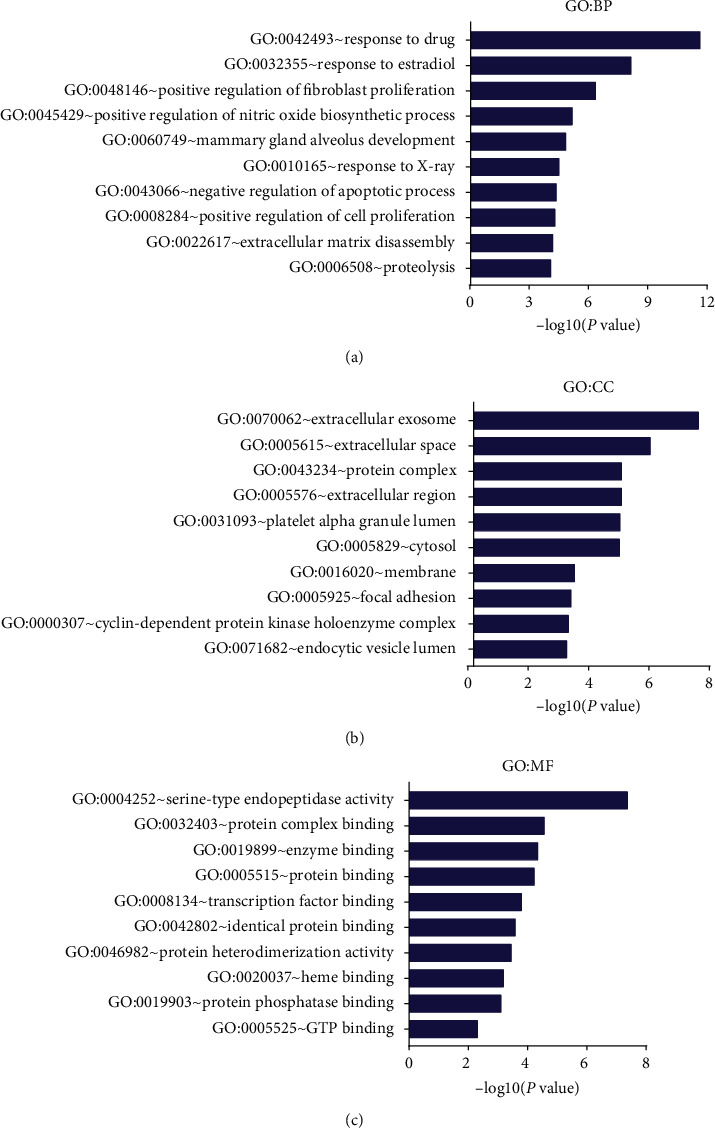
Gene ontology analyses of the therapeutic target genes of *Scutellariae Radix* in the treatment of hepatocellular carcinoma. (a) Biological process (BP), (b) cellular component (CC), and (c) molecular function (MF).

**Figure 6:**
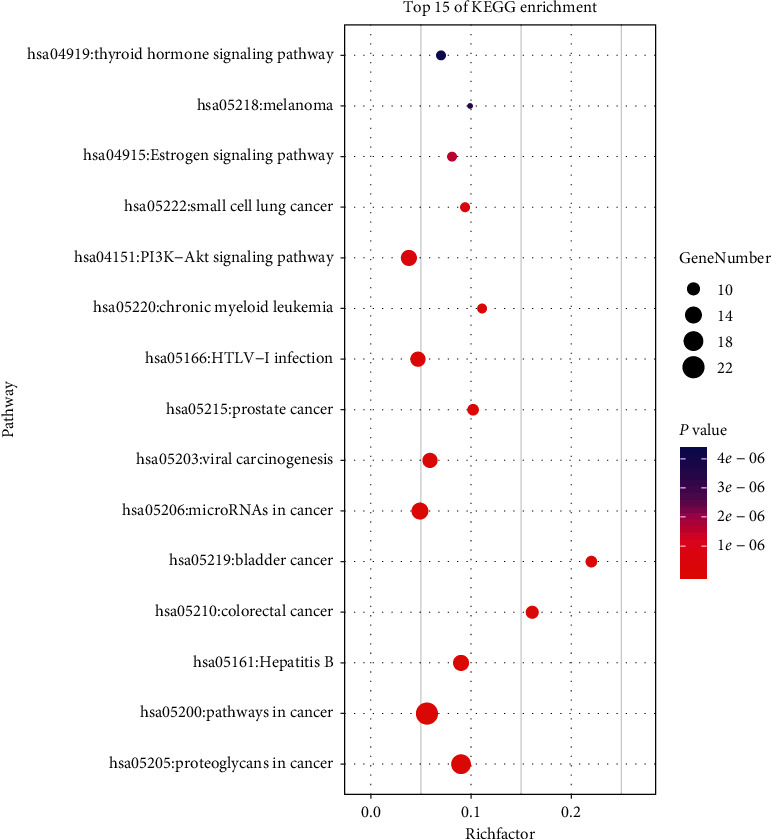
KEGG pathway enrichment analyses of therapeutic target genes of *Scutellariae Radix* in the treatment of hepatocellular carcinoma.

The GO and KEGG pathways of the associated targets were analyzed using the DAVID 6.8 platform to identify the biological function and signaling pathways of 50 candidate targets of SR for HCC treatment. A total of 131 biological process (BP) terms, 23 cellular component (CC) terms, and 35 molecular function (MF) terms met the screening criteria of count ≥ 2 and *P* ≤ 0.05. The detailed information of GO terms is listed in Supplementary Table S4. The top 10 remarkably enriched terms in BP, CC, and MF classification are presented in Figure 5. In the light of false discovery rate (FDR) < 0.05, 13 GO terms, including 5 BP, 6 CC, and 2 MF terms, were found, suggesting that SR might regulate fibroblast proliferation and nitric oxide biosynthetic process via the serine-type endopeptidase activity and protein complex binding in the cytosol, extracellular region, and extracellular exosomes to produce the treatment action on HCC. The KEGG pathway analysis of 50 candidate targets was performed using the DAVID platform. A total of 59 pathways were distinctly enriched (*P* < 0.05), and the detailed information is listed in Supplementary Table S4. With the screening criteria of *P* < 0.01 and FDR < 0.01, top 15 remarkably enriched pathways were obtained (Figure 6). The following pathways were primarily enriched: biochemical substances involved with cancer (proteoglycans and microRNAs); pathways in cancer; human diseases associated with cancer (colorectal cancer, bladder cancer, prostate cancer, chronic myeloid leukemia, small cell lung cancer, and melanoma); diseases linked to virus infection (hepatitis B, viral carcinogenesis, and human. T-cell leukemia virus type I infection); and signal transduction pathways (PI3K-Akt, estrogen, and thyroid hormone signaling pathways).

### 3.6. Integrated Network Construction

**Figure 7:**
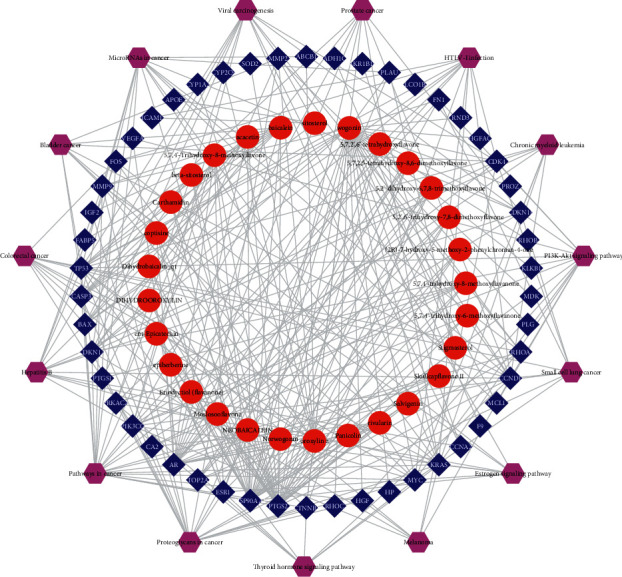
Compound-target-signaling pathway network of *Scutellariae Radix* on hepatocellular carcinoma. Red ellipse stands for candidate active compound, blue diamond stands for compound target, and purple hexagon stands for pathway.

Medicine plays a pharmacological role through an integrative molecular interaction network [27]. In our research, a compound-target-pathway network of SR for HCC treatment was constructed (Figure 7). This network was made up of 94 nodes (29 for components, 50 for targets, and 15 for pathways). This integrative network indicated that the antitumor effect of SR on HCC might be attributed to the active components (wogonin, oroxylin A, and baicalein) acting on candidate protein targets (PTGS2, HSP90AA1, AR, TP53, GTPase KRas (KRAS), phosphatidylinositol 4,5-bisphosphate 3-kinase catalytic subunit gamma isoform (PIK3CG), and CCND1) that regulate key pathways (pathways in cancer, proteoglycans or microRNAs in cancer, hepatitis B, and PI3K-Akt signaling pathway) to affect the survival of HCC cells.

## 4. Discussion

HCC is a disease with high mortality but without effective drugs for most patients. Herbal medicine has been used to treat human diseases for thousands of years and considered a real treasure source for drug development. Network pharmacology is a new discipline associated with pharmacy, medical science, computer science, and bioinformatics. It provides a platform related to the concept of systems biology, which is suitable for CHM research [28]. Therefore, new drug information should be developed through drug-target-disease network analysis.

Herbal medicine is a complex system composed of multiple compounds. The compounds with suitable pharmacokinetic properties (OB ≥ 30% and DL index ≥ 0.18) were thought to be potential bioactive compounds. The targets of these potential bioactive components were mapped with HCC targets to acquire the targets of SR for HCC treatment. The compound-compound target network was constructed to explore which components of SR act on their corresponding targets to treat HCC. These results revealed that wogonin, oroxylin A, and baicalein were three components with high degrees, and they were consistent with previous findings [29], which suggested that wogonin, oroxylin A, baicalein, and their glucuronide/sulfate-conjugated metabolites were the main active components in the liver and tumors. In general, baicalein is one of the most significant components of SR and has been developed into a new drug to treat hepatitis in clinical settings. Reviews [30] and network pharmacology [17] studies on the action of baicalein against HCC have been reported. Baicalein decreased the expression of AKT, beta-catenin (CTNNB1), and cyclin D1 (CCND1), leading to the cell cycle arrest [31] and inhibiting the proliferation of HCC cells by suppressing the PI3K-Akt pathway [32]. By comparison, fewer studies on oroxylin A and wogonin have been performed. Notably, about 34%–63% of baicalin was methylated to oroxylin A in various organs during absorption. Oroxylin A triggered the apoptosis of HCC cell line HepG2 by inactivating AKT signaling [33] or regulating glucose metabolism [34]. Wogonin can regulate the activation of hepatic stellate cells and their apoptosis to attenuate liver fibrosis, which is an important pathological process in the progression of liver cancer [35]. Wogonin inhibits the cell cycle progression and migration [36] and induces the apoptosis of HCC cells [37]. It is an attractive new anticancer and antihepatitis B virus [38] drug candidate and is being developed with other drugs as a targeted therapy for HCC [39, 40]. The major active components, including wogonin, oroxylin A, and baicalein, in SR can inhibit the proliferation of HCC cells by regulating the PI3K-Akt signaling pathway [32, 33]. Oroxylin A and wogonin have a synergistic effect when they are combined with 5-fluorouracil in HCC cells [41–43]. PTGS2, HSP90AA1, and AR were the top three high-degree targets. PTGS2 can serve as one of the biomarkers on account of aberrant methylation for the precise treatment of HCC [44]. Notably, ketoconazole is regarded as a potential therapeutic choice for HCC treatment by acting on PTGS2 [45]. HSP90AA1 is a candidate diagnostic and prognostic biomarker for HCC [46]. HSP90 can promote glycolysis and attenuate the apoptosis of HCC cells by affecting pyruvate kinase M2 [47]. AR has been considered in relation to the pathogenesis of HCC, which is a male-dominant cancer. A high mRNA of AR is frequently involved with a better survival of HCC [48].

The compound-compound target network indicated the potential direct protein targets. The PPI network revealed the relationship between the candidate targets and other human targets and suggested the change in potential biological functions through the PPI. With topology analysis, the core targets, including candidate protein targets and human other targets, were found. The degree of the nodes suggested their significance and the relation with other nodes. The core targets were the nodes whose degree was more than twice the mean degree of the nodes in the PPI network. These targets included candidate protein targets (TP53, CCND1, MYC, VEGFA, CTNNB1, RHOA, CCNA2, AR, and HSP90AA1) and human other targets (CDK2, CDH1, CDK1, EP300, and JUN). The mutation of TP53 (cellular tumor antigen p53) and CCND1 (G1/S-specific cyclin-D1), serving as tumor suppressors associated with cell cycle regulation, is regarded as drivers of HCC development [49]. VEGFA, a growth factor active in angiogenesis and endothelial cell growth, can increase endothelial cell proliferation and reduce the apoptosis of blood vessels. Myc protooncogene protein is a transcription factor that promotes VEGFA production and subsequent angiogenesis. The transforming protein RhoA is a small GTPase that is frequently upregulated in HCC linked to poor prognosis [50]. CDK1 and CDK2 play an essential role in the regulation of the cell cycle with multiple interphase cyclins. CCNA2 interferes with the G1/S and G2/M phases of the cell cycle by activating CDK1 and CDK2. EP300, acting as a histone acetyltransferase, regulates transcription via chromatin remodeling and is related to the poor prognosis of HCC [51]. JUN function as a transcription factor specifically increased in HBV-related HCC [52]. In summary, these targets are mostly involved with the cell proliferation, metastasis, and survival of patients with HCC.

**Figure 8:**
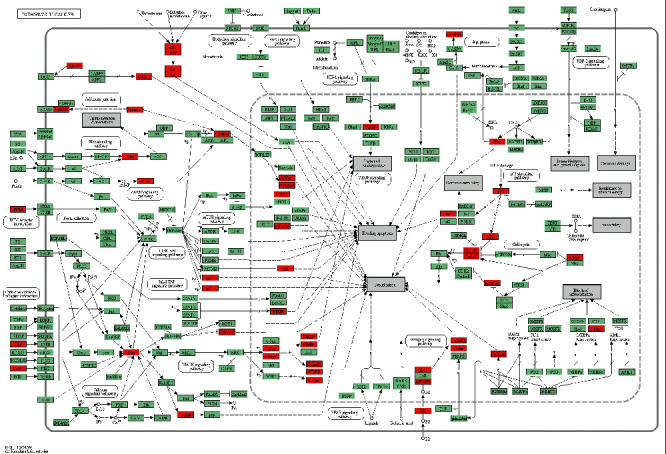
Pathways in cancer regulated by *Scutellariae Radix* on hepatocellular carcinoma.

GO function annotation was performed to acquire the biological information from BP, CC, and MF aspects. BP, CC, and MF, respectively, display a series of events produced by the orderly combination of molecules, cellular localization, and molecular activity of the target proteins. In this study, BP involved events such as responses to drugs or estradiol, regulation of fibroblast proliferation, regulation of nitric oxide biosynthesis, and mammary gland alveolus development. CC indicated that these candidate targets were mainly localized in the extracellular region and the protein complex. MF showed a serine-type endopeptidase activity and protein complex binding. The KEGG pathway is a set of pathway maps depicting our understanding on molecular interactions and relationship networks. The results of the KEGG pathway enrichment showed that the candidate targets were remarkably enriched on pathways in cancer, hepatitis B, viral carcinogenesis, PI3K-Akt signaling pathway, and so on. Twenty-two targets were enriched in pathways in cancer (Figure 8), which contained AR signaling pathway, PI3K-Akt signaling pathway, MAPK signaling pathway, estrogen signaling pathway, p53 signaling pathway, and cell cycle. Among these targets, TP53, KRAS, MYC, and CCND1 were the vital targets existing in the crosstalk with other signaling pathways that regulate the cycle, proliferation, and apoptosis of cancer cells. Moreover, the AR and estrogen signaling pathways indicated the significant role of hormones in HCC, as HCC occurs in men more often than it does in women. Hepatitis B is a predominant reason leading to HCC, and the therapeutic effect of SR on HCC may be associated with its antiviral effect. Thirteen potential targets were enriched in the PI3K-Akt signaling pathway, which plays a crucial role in the occurrence and development of HCC [53].

Network pharmacology is an essential field providing a vital approach for ascertaining novel targets for rational drug discovery. Different from conventional drug discovery approaches which are commonly based on specific targeting of single proteins, network pharmacology focuses on drug targets concerning myriads of proteins involved in a disease. Network pharmacology helps to build pragmatic network models and predict drug targets on the basis of public databases. Additionally, it also facilitates the establishment of drug-target-disease network models using bioinformatics and high-throughput screening. The application of network pharmacology for the design of potent anticancer drug combinations has been demonstrated [54]. Therefore, network pharmacology approaches may revolutionize future drug discovery and development.

## 5. Conclusion

In the current study, network pharmacology was applied to characterize the mechanism of SR for HCC treatment. Integrated compound-target-pathway network analysis displayed the candidate active components (such as wogonin, oroxylin A, and baicalein) exerted their antitumor effect by regulating pathways in cancer, hepatitis B, viral carcinogenesis, PI3K-Akt signaling, and so on. This holistic approach can provide novel insights into the mechanistic study and therapeutic drug development of herbal medicine on HCC.

## Figures and Tables

**Table 1:** Information of the candidate bioactive components of *Scutellariae Radix*.

Molecule ID	Molecule name	MW	OB (%)	DL	Herb
MOL000073	Ent-epicatechin	290.29	48.96	0.24	*Scutellariae Radix*
MOL000173	Wogonin	284.28	30.68	0.23	*Scutellariae Radix*
MOL000228	(2R)-7-hydroxy-5-methoxy-2-phenylchroman-4-one	270.30	55.23	0.20	*Scutellariae Radix*
MOL000358	Beta-sitosterol	414.79	36.91	0.75	*Scutellariae Radix*
MOL000359	Sitosterol	414.79	36.91	0.75	*Scutellariae Radix*
MOL000449	Stigmasterol	412.77	43.83	0.76	*Scutellariae Radix*
MOL000525	Norwogonin	270.25	39.40	0.21	*Scutellariae Radix*
MOL000552	5,2′-Dihydroxy-6,7,8-trimethoxyflavone	344.34	31.71	0.35	*Scutellariae Radix*
MOL001458	Coptisine	320.34	30.67	0.86	*Scutellariae Radix*
MOL001490	bis[(2S)-2-ethylhexyl] benzene-1,2-dicarboxylate	390.62	43.59	0.35	*Scutellariae Radix*
MOL001506	Supraene	410.80	33.55	0.42	*Scutellariae Radix*
MOL001689	Acacetin	284.28	34.97	0.24	*Scutellariae Radix*
MOL002714	Baicalein	270.25	33.52	0.21	*Scutellariae Radix*
MOL002879	Diop	390.62	43.59	0.39	*Scutellariae Radix*
MOL002897	Epiberberine	336.39	43.09	0.78	*Scutellariae Radix*
MOL002908	5,8,2′-Trihydroxy-7-methoxyflavone	300.28	37.01	0.27	*Scutellariae Radix*
MOL002909	5,7,2,5-Tetrahydroxy-8,6-dimethoxyflavone	376.34	33.82	0.45	*Scutellariae Radix*
MOL002910	Carthamidin	288.27	41.15	0.24	*Scutellariae Radix*
MOL002911	2,6,2′,4′-Tetrahydroxy-6′-methoxychaleone	302.30	69.04	0.22	*Scutellariae Radix*
MOL002913	Dihydrobaicalin_qt	272.27	40.04	0.21	*Scutellariae Radix*
MOL002914	Eriodyctiol (flavanone)	288.27	41.35	0.24	*Scutellariae Radix*
MOL002915	Salvigenin	328.34	49.07	0.33	*Scutellariae Radix*
MOL002917	5,2′,6′-Trihydroxy-7,8-dimethoxyflavone	330.31	45.05	0.33	*Scutellariae Radix*
MOL002925	5,7,2′,6′-Tetrahydroxyflavone	286.25	37.01	0.24	*Scutellariae Radix*
MOL002926	Dihydrooroxylin A	286.30	38.72	0.23	*Scutellariae Radix*
MOL002927	Skullcapflavone II	374.37	69.51	0.44	*Scutellariae Radix*
MOL002928	Oroxylin A	284.28	41.37	0.23	*Scutellariae Radix*
MOL002932	Panicolin	314.31	76.26	0.29	*Scutellariae Radix*
MOL002933	5,7,4′-Trihydroxy-8-methoxyflavone	300.28	36.56	0.27	*Scutellariae Radix*
MOL002934	Neobaicalein	374.37	104.34	0.44	*Scutellariae Radix*
MOL002937	Dihydrooroxylin	286.30	66.06	0.23	*Scutellariae Radix*
MOL008206	Moslosooflavone	298.31	44.09	0.25	*Scutellariae Radix*
MOL010415	11,13-Eicosadienoic acid, methyl ester	322.59	39.28	0.23	*Scutellariae Radix*
MOL012245	5,7,4′-Trihydroxy-6-methoxyflavanone	302.30	36.63	0.27	*Scutellariae Radix*
MOL012246	5,7,4′-Trihydroxy-8-methoxyflavanone	302.30	74.24	0.26	*Scutellariae Radix*
MOL012266	Rivularin	344.34	37.94	0.37	*Scutellariae Radix*
MOL002776	Baicalin	446.39	40.12	0.75	*Scutellariae Radix*

## Data Availability

The data used to support the findings of this study are included within the article and the supplementary materials.

## Supplementary Materials

Supplementary Materials Supplementary Table S1: targets of candidate active components in *Scutellariae Radix*. Supplementary Table S2: the detailed information of 566 HCC-related human genes. Supplementary Table S3: candidate targets of *Scutellariae Radix* against HCC. Supplementary Table S4: results of GO and KEGG pathway analysis using DAVID database.
